# Supplementary material for: Caffeic Acid Phosphanium Derivatives: Potential Selective Antitumor, Antimicrobial and Antiprotozoal Agents
Source: Int J Mol Sci. 2024 Jan 18;25(2):1200. doi: 10.3390/ijms25021200 (PMC10817097; doi:10.3390/ijms25021200)
Supplement: Supplementary file 1 [file ijms-25-01200-s001.zip › ijms-2764762-supplementary.pdf]

Supporting Information

Caffeic Acid Phosphanium Derivatives: Potential Selective Antitumor, Antimicrobial and Antiprotozoal Agents

Miloš Lukáč [1], Lívia Slobodníková [2], Martin Mrva [3], Aneta Dušeková [3], Mária Garajová [3], Martin Kello [4], Dominika Šebová [4], Martin Pisárčik [1], Marián Kojnok [1], Andrej Vrták [1], Elena Kurin [5] and Silvia Bittner Fialová [5,*]

[1] Department of Chemical Theory of Drugs, Faculty of Pharmacy, Comenius University Bratislava, Odbojárov 10, 832 32 Bratislava, Slovakia; lukac@fpharm.uniba.sk (M.L.); pisarcik@fpharm.uniba.sk (M.P.); mariankojnok219@gmail.com (M.K.); andrej1616@gmail.com (A.V.)
[2] Institute of Microbiology, Faculty of Medicine, Comenius University Bratislava, University Hospital in Bratislava, Sasinkova 4, 811 08 Bratislava, Slovakia; livia.slobodnikova@fmed.uniba.sk
[3] Department of Zoology, Faculty of Natural Sciences, Comenius University Bratislava, Mlynská Dolina, Ilkovičova 6, 842 15 Bratislava, Slovakia; martin.mrva@uniba.sk (M.M.); aneta.dusekova@gmail.com (A.D.); maria.garajova@uniba.sk (M.G.)
[4] Department of Pharmacology, Faculty of Medicine, P.J. Šafárik University, Trieda SNP 1, 040 11 Košice, Slovakia; martin.kello@upjs.sk (M.K.); dominika.sebova@student.upjs.sk (D.Š.)
[5] Department of Pharmacognosy and Botany, Faculty of Pharmacy, Comenius University Bratislava, Odbojárov 10, 832 32 Bratislava, Slovakia; elena.kurin@uniba.sk
* Correspondence: fialova@fpharm.uniba.sk; Tel.: +421-250-117-206

Table of Contents

Figure S1. Chemical structure of CAP 6 (n = 3), CAP 8 (n = 4), CAP 10 (n = 5), CAP 12 (n = 6)................3

Figure S2. 13C NMR data of CAP 6 (in CDCl3) 4

Figure S3. ^{1}H NMR data of CAP 6 (in $CDCl_3$) 5

Figure S4. ^{31}P NMR data of CAP 6 (in $CDCl_3$)..6

Figure S5. 13C NMR data of CAP 8 (in CDCl3) 7

Figure S6. ^{1}H NMR data of CAP 8 (in $CDCl_3$) 8

Figure S7. ^{31}P NMR data of CAP 8 (in $CDCl_3$)..9

Figure S8. ^{13}C NMR data of CAP 10 (in $CDCl_3$) 10

Figure S9. ^{1}H NMR data of CAP 10 (in $CDCl_3$) 11

Figure S10. ^{31}P NMR data of CAP 10 (in $CDCl_3$)..12

Figure S11. ^{13}C NMR data of CAP 12 (in $CDCl_3$) 13

Figure S12. ^{1}H NMR data of CAP 12 (in $CDCl_3$) 14

Figure S13. ^{31}P NMR data of CAP 12 (in $CDCl_3$)..15

Figure S14. ^{13}C NMR data of CAP 6 ester (in $CDCl_3$)..16

Figure S15. ^{1}H NMR data of CAP 6 ester (in $CDCl_3$)..17

Figure S16. ^{13}C NMR data of CAP 8 ester (in $CDCl_3$)..18

Figure S17. ^{1}H NMR data of CAP 8 ester (in $CDCl_3$)..19

Figure S18. ^{13}C NMR data of CAP 10 ester (in $CDCl_3$)..20

Figure S19. ^{1}H NMR data of CAP 10 ester (in $CDCl_3$)..21

Figure S20. ^{13}C NMR data of CAP 12 ester (in $CDCl_3$)..22

Figure S21. ^{1}H NMR data of CAP 12 ester (in $CDCl_3$)..23

Figure S22. HPLC-DAD data of CAP 6 (60% H_2O + 0.05% TFA/40% ACN + 0.05% TFA) at λ = 254 nm24

Figure S23. HPLC-DAD data of CAP 8 (60% H_2O + 0.05% TFA/40% ACN + 0.05% TFA) at λ = 254 nm25

Figure S24. HPLC-DAD data of CAP 10 (60% H_2O + 0.05% TFA/40% ACN + 0.05% TFA) at λ = 254 nm26

Figure S25. HPLC-DAD data of CAP 12 (60% H_2O + 0.05% TFA/40% ACN + 0.05% TFA) at λ = 254 nm27

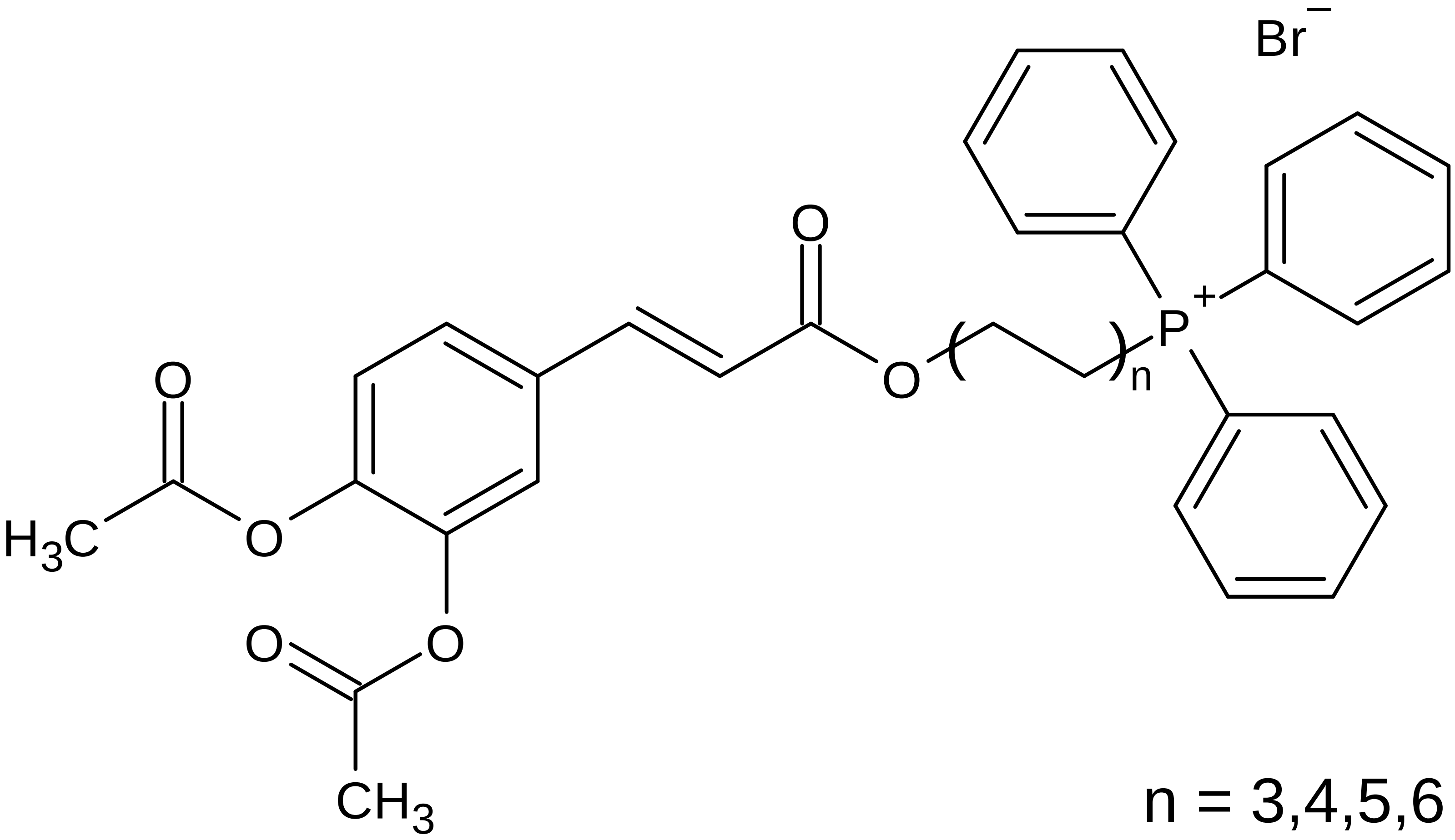

Figure S1. Chemical structure of CAP 6 (n = 3), CAP 8 (n = 4), CAP 10 (n = 5), CAP 12 (n = 6)

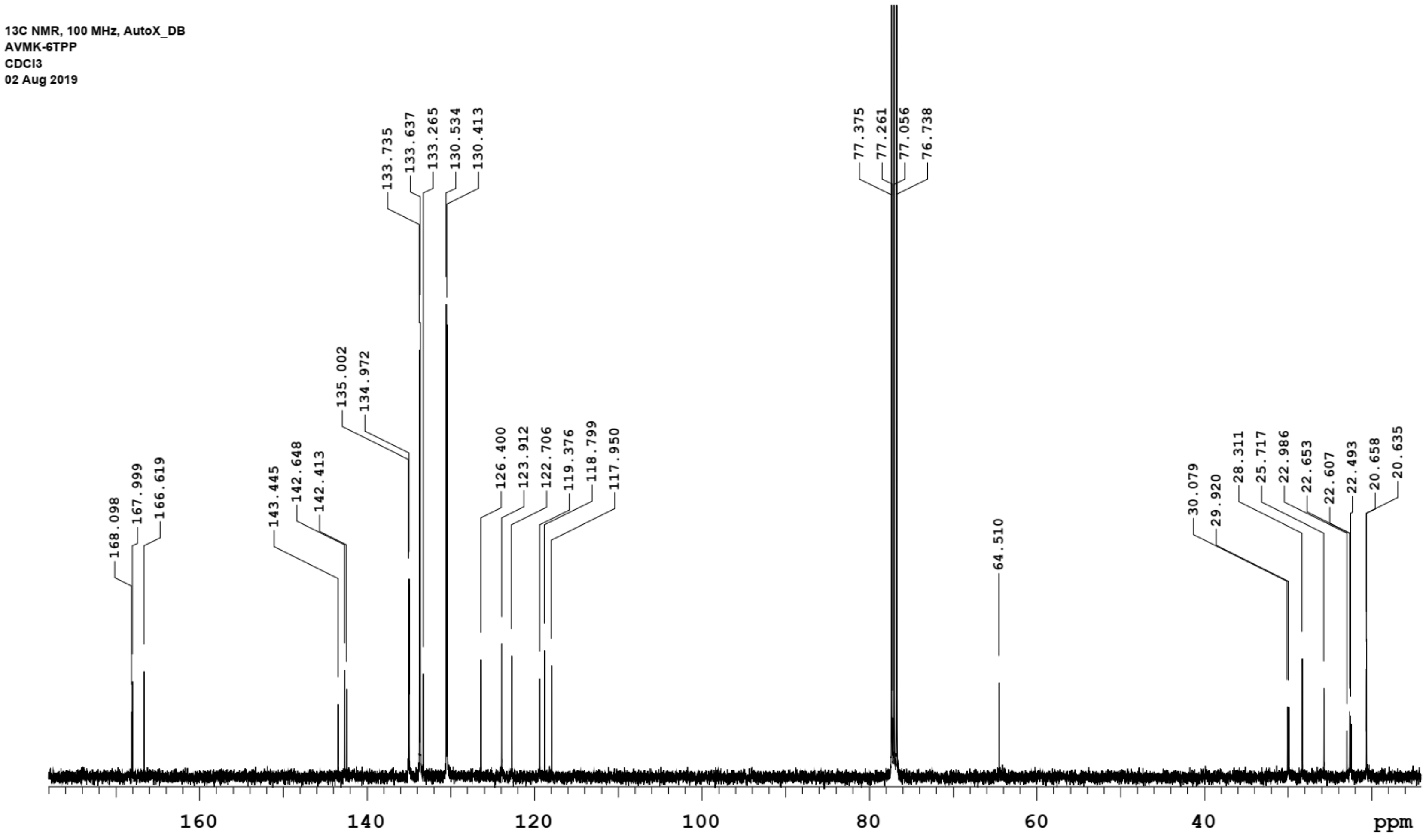

Figure S2. 13C NMR data of CAP 6 (in CDCl3)

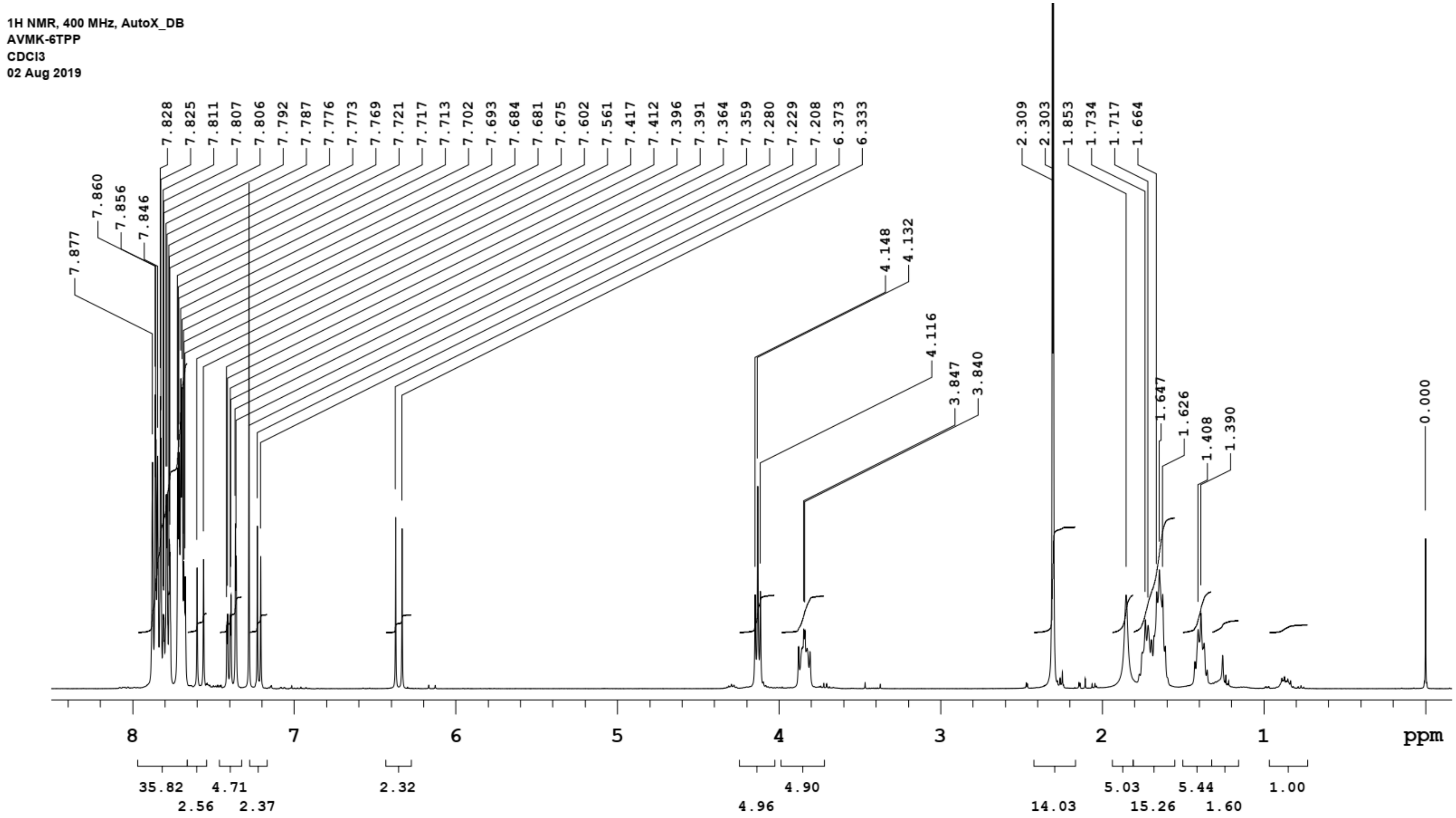

Figure S3. ^{1}H NMR data of CAP 6 (in $CDCl_3$)

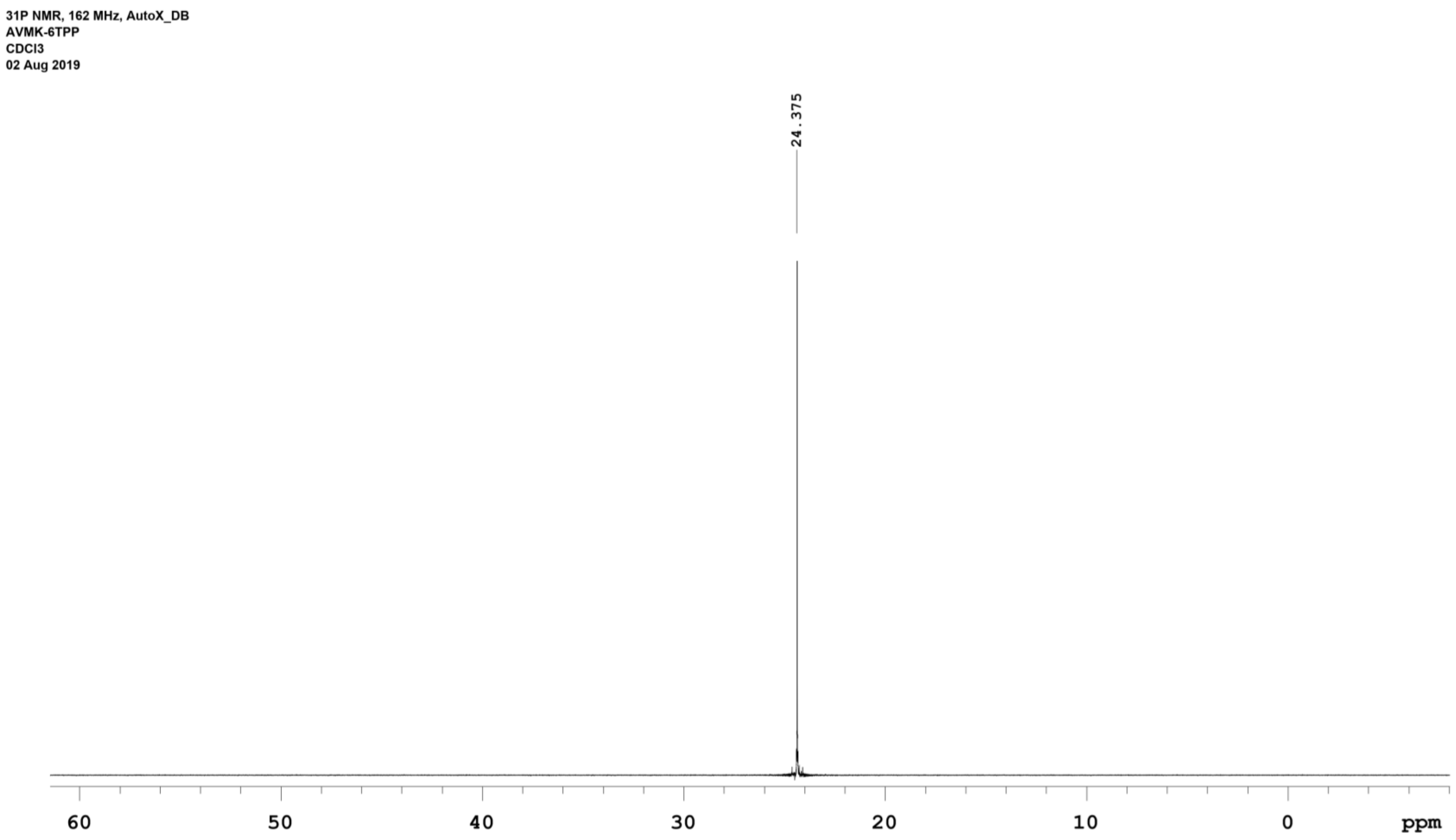

Figure S4. ^{31}P NMR data of CAP 6 (in $CDCl_3$)

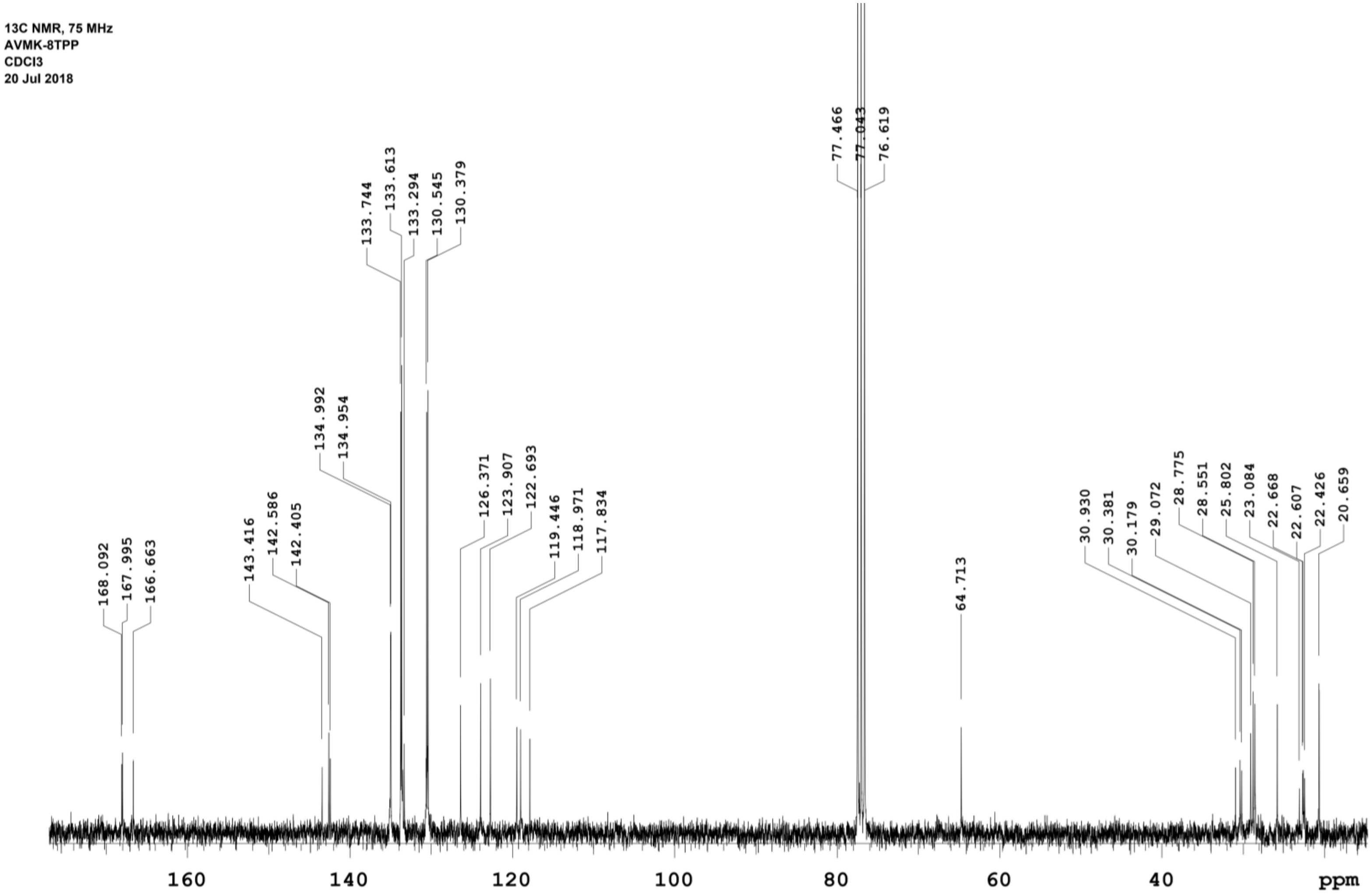

Figure S5. 13C NMR data of CAP 8 (in CDCl3)

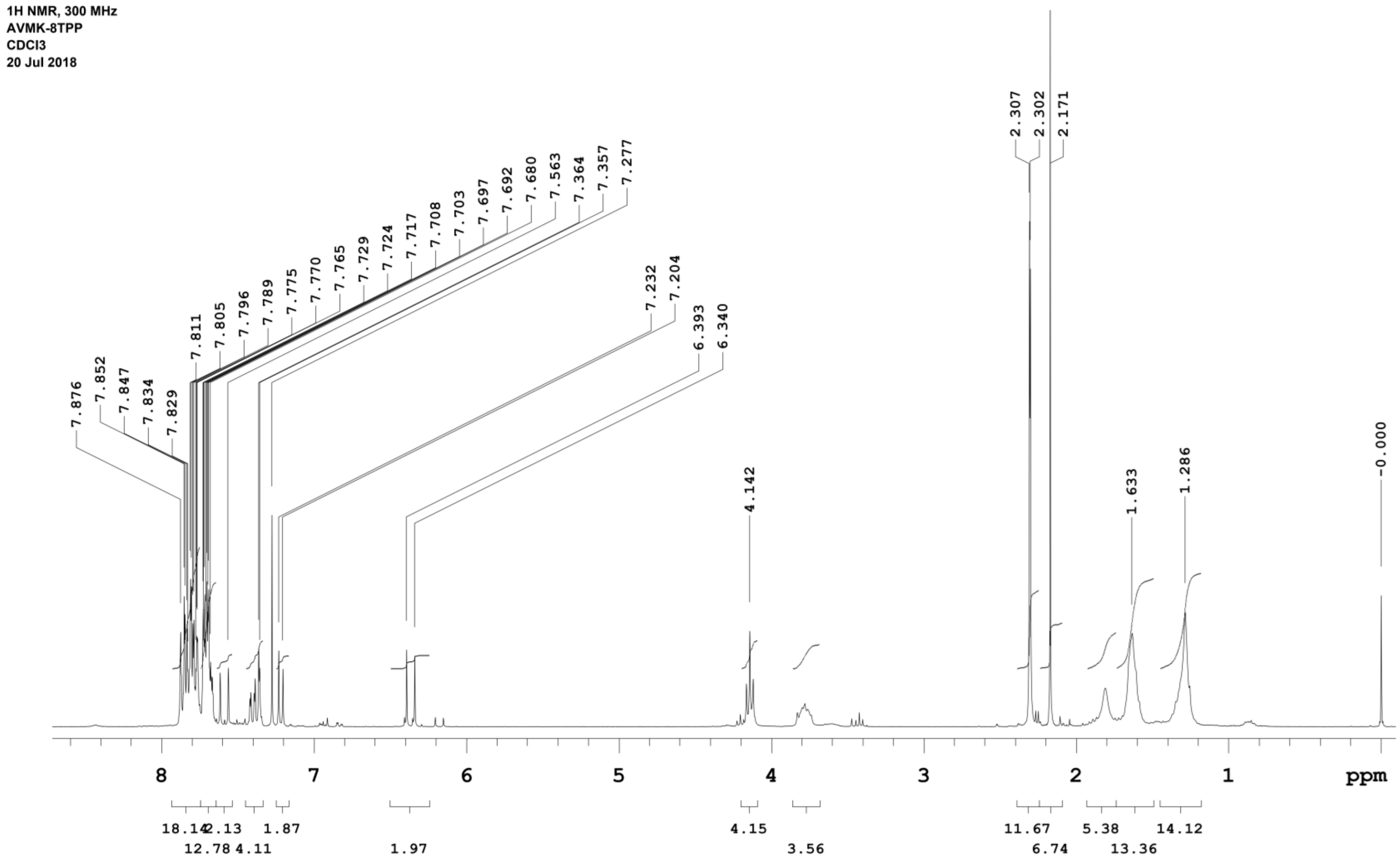

Figure S6. ^{1}H NMR data of CAP 8 (in $CDCl_3$)

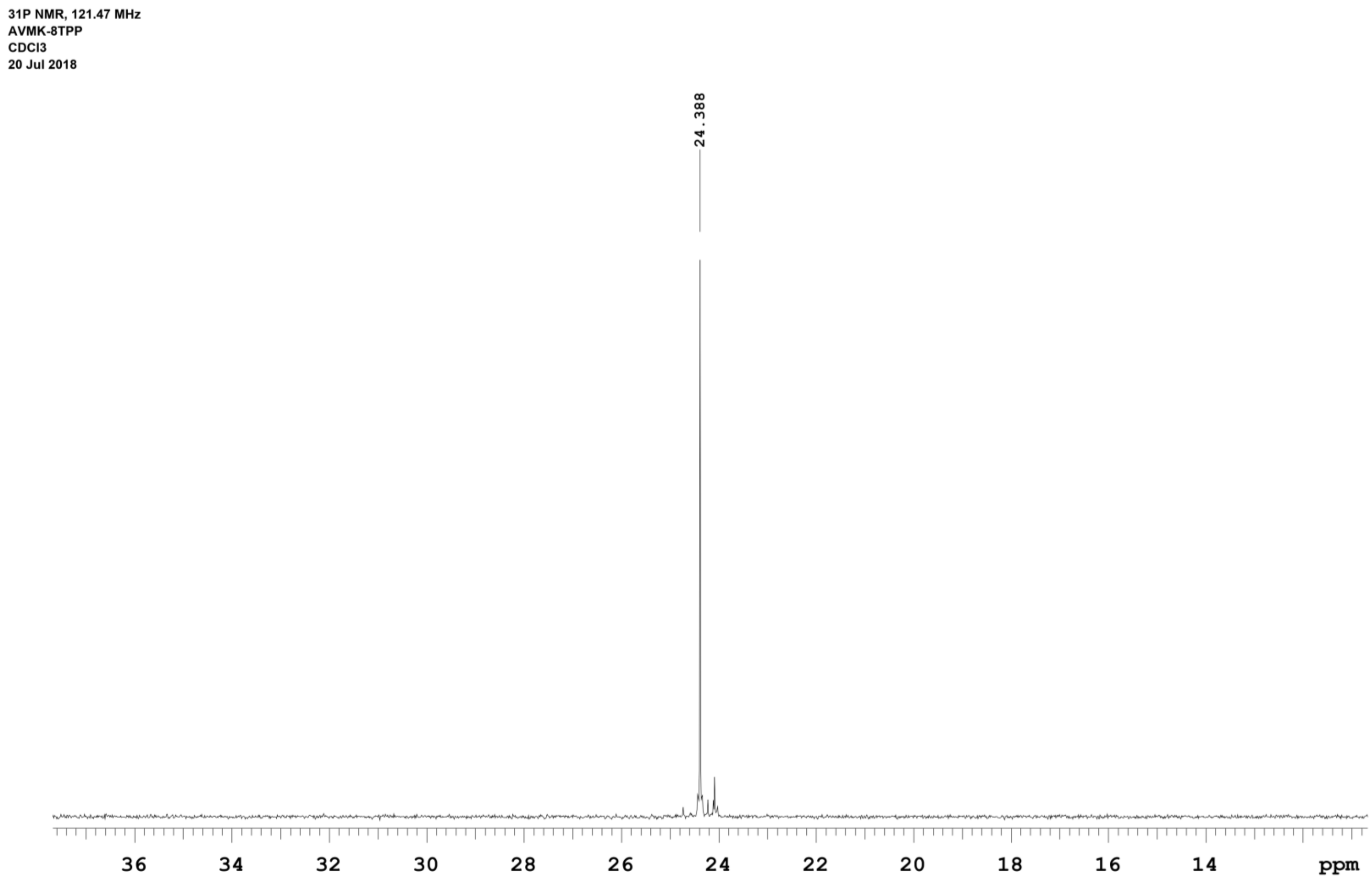

Figure S7. ^{31}P NMR data of CAP 8 (in $CDCl_3$)

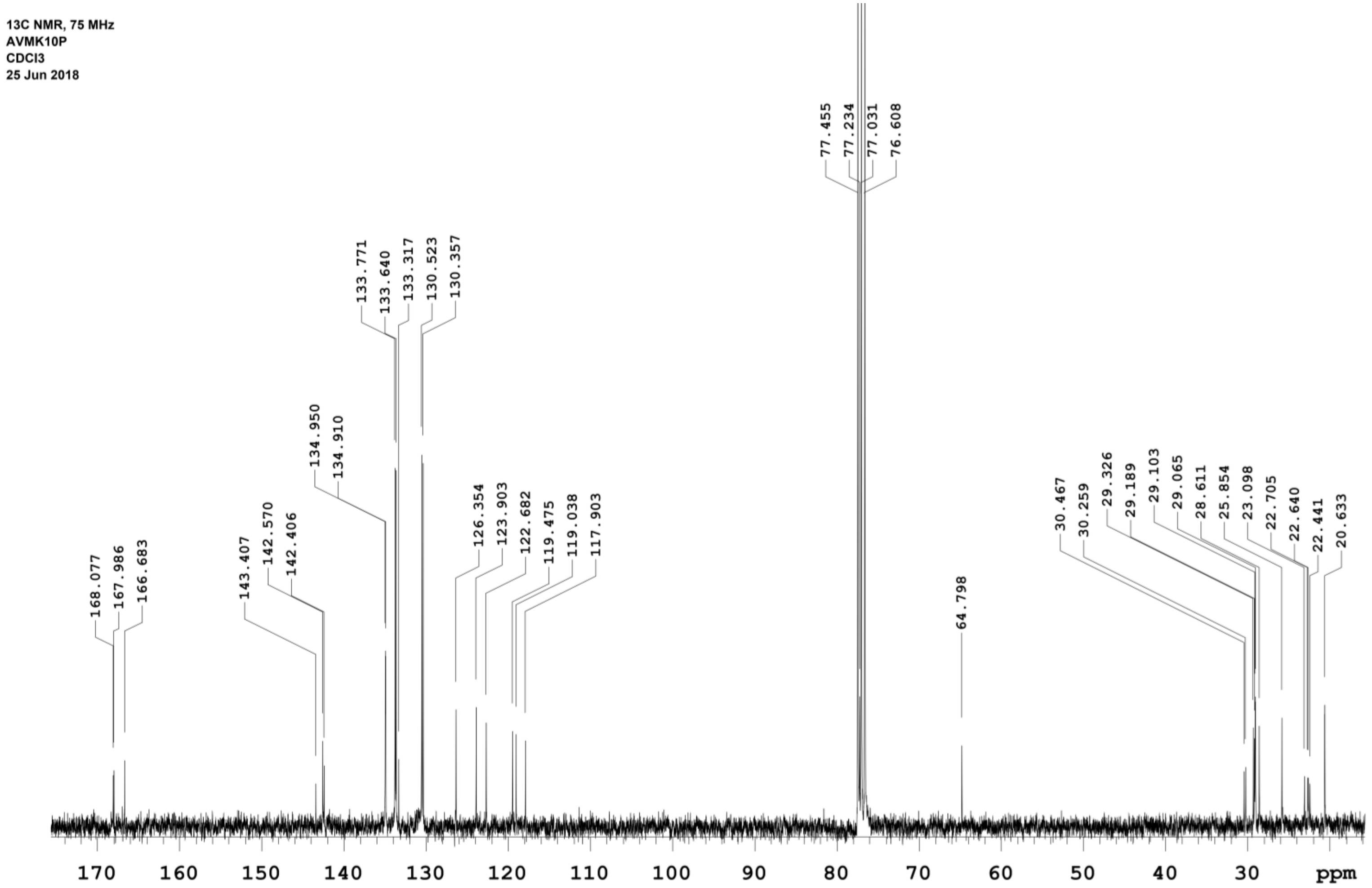

Figure S8. ^{13}C NMR data of CAP 10 (in $CDCl_3$)

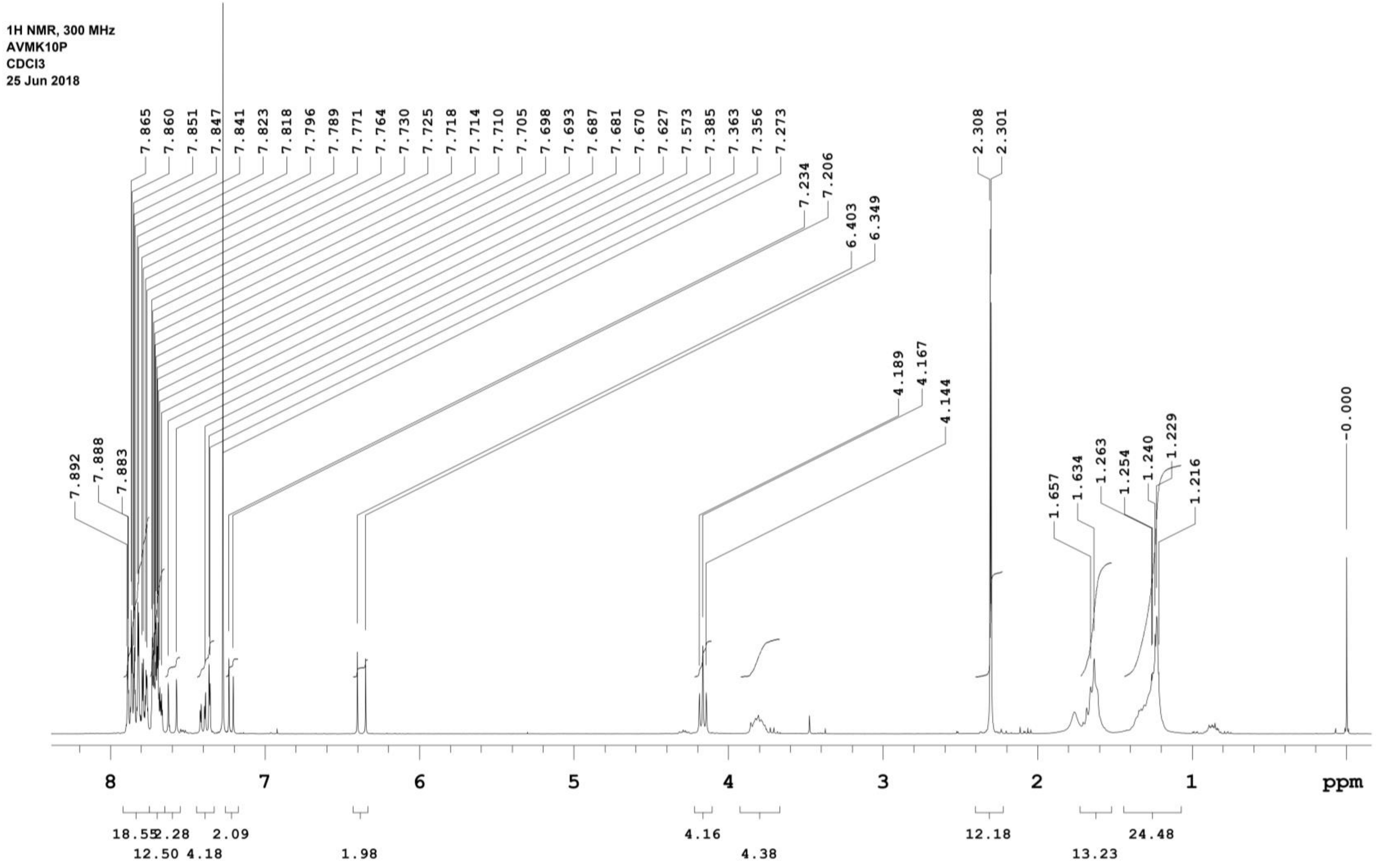

Figure S9. ^{1}H NMR data of CAP 10 (in $CDCl_3$)

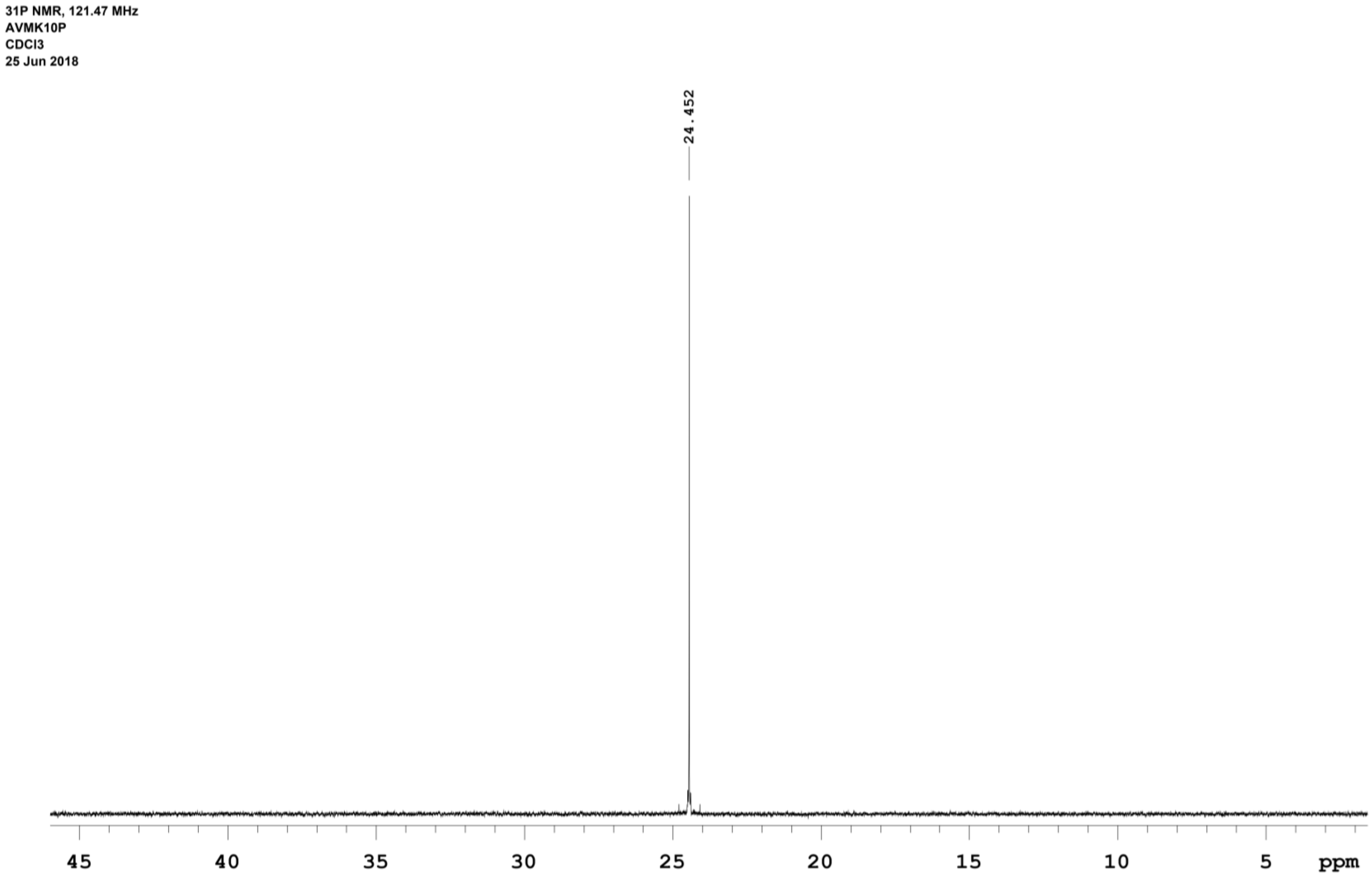

Figure S10. ^{31}P NMR data of CAP 10 (in $CDCl_3$)

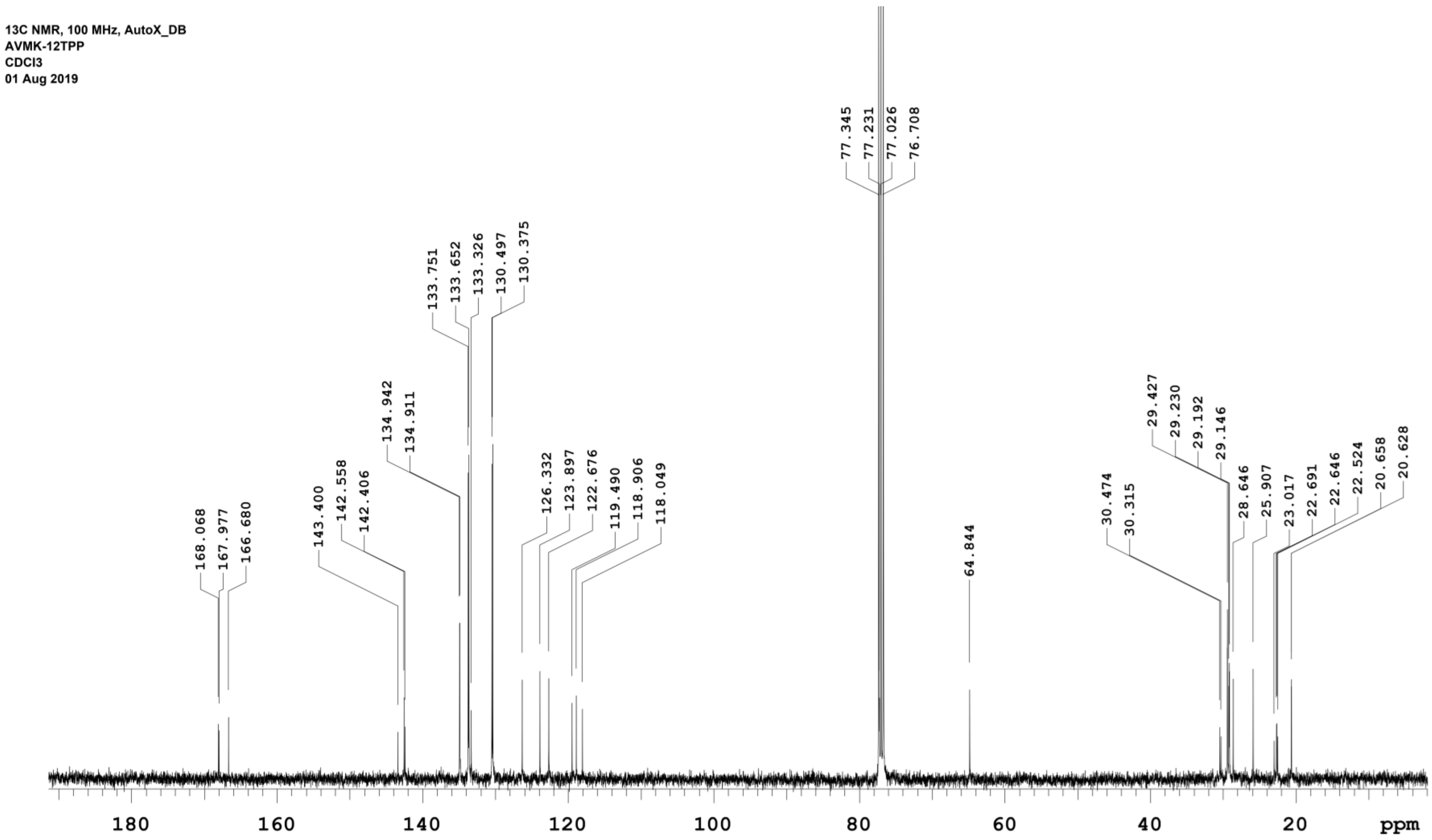

Figure S11. ^{13}C NMR data of CAP 12 (in $CDCl_3$)

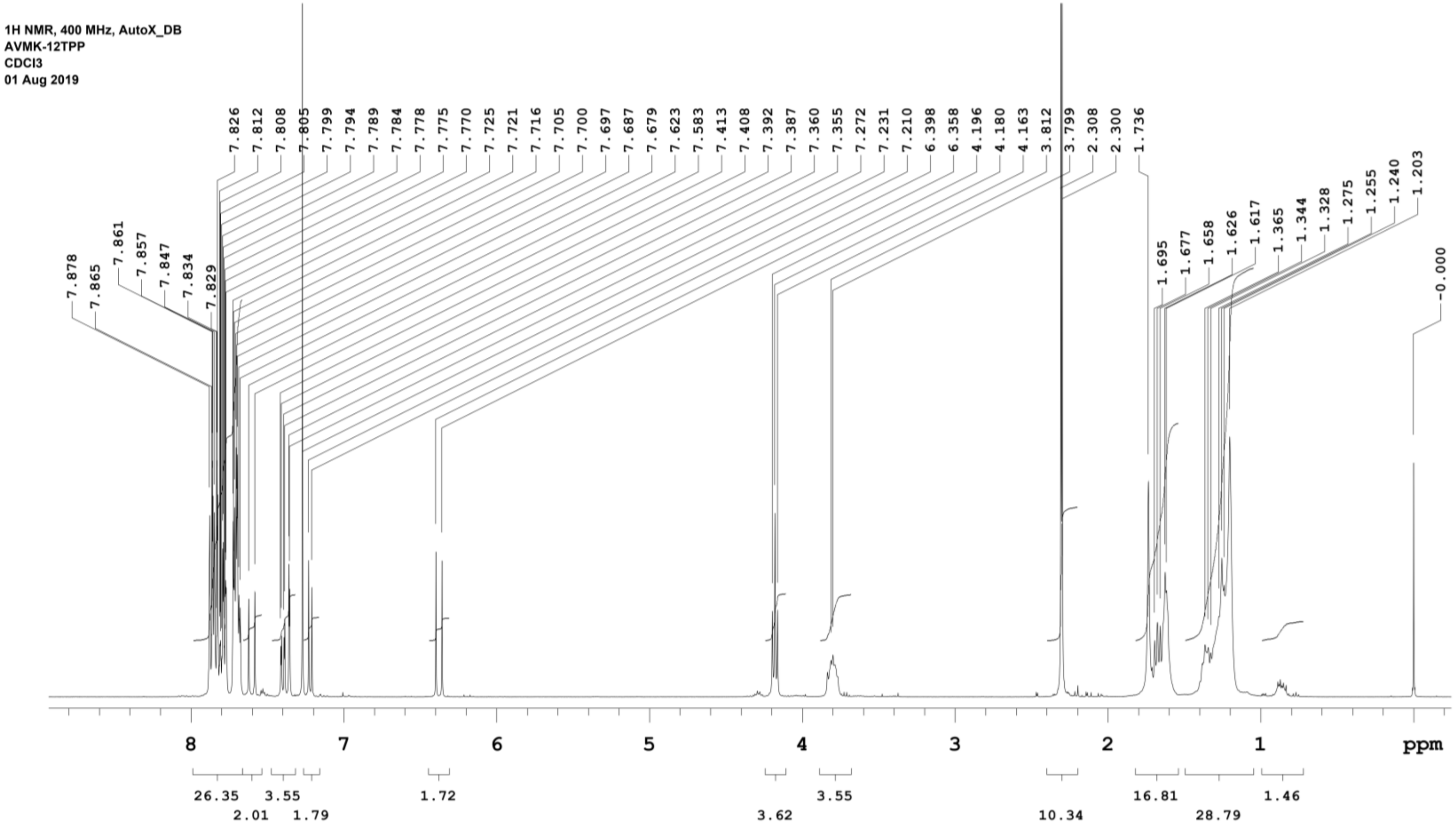

Figure S12. ^{1}H NMR data of CAP 12 (in $CDCl_3$)

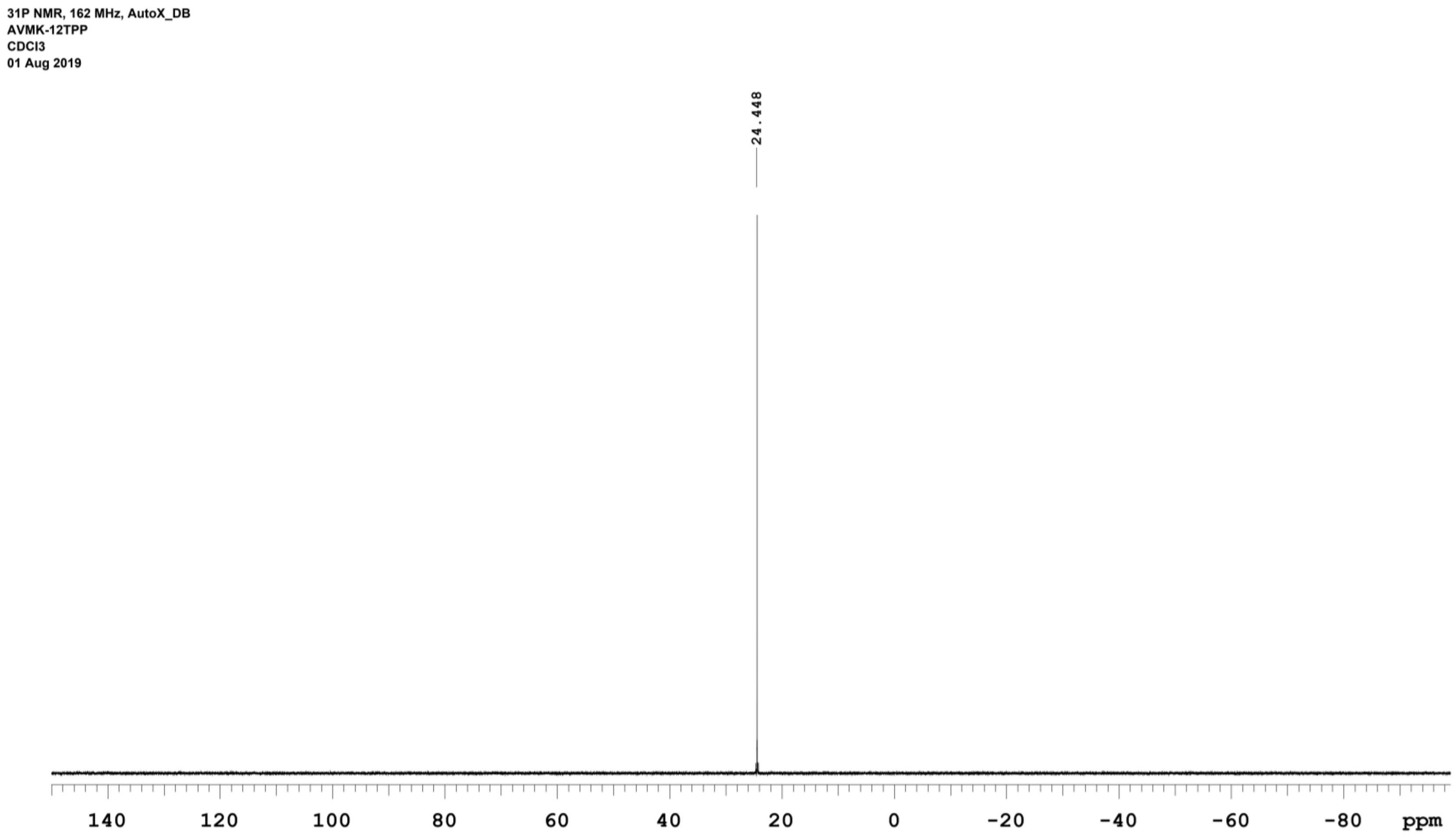

Figure S13. ^{31}P NMR data of CAP 12 (in $CDCl_3$)

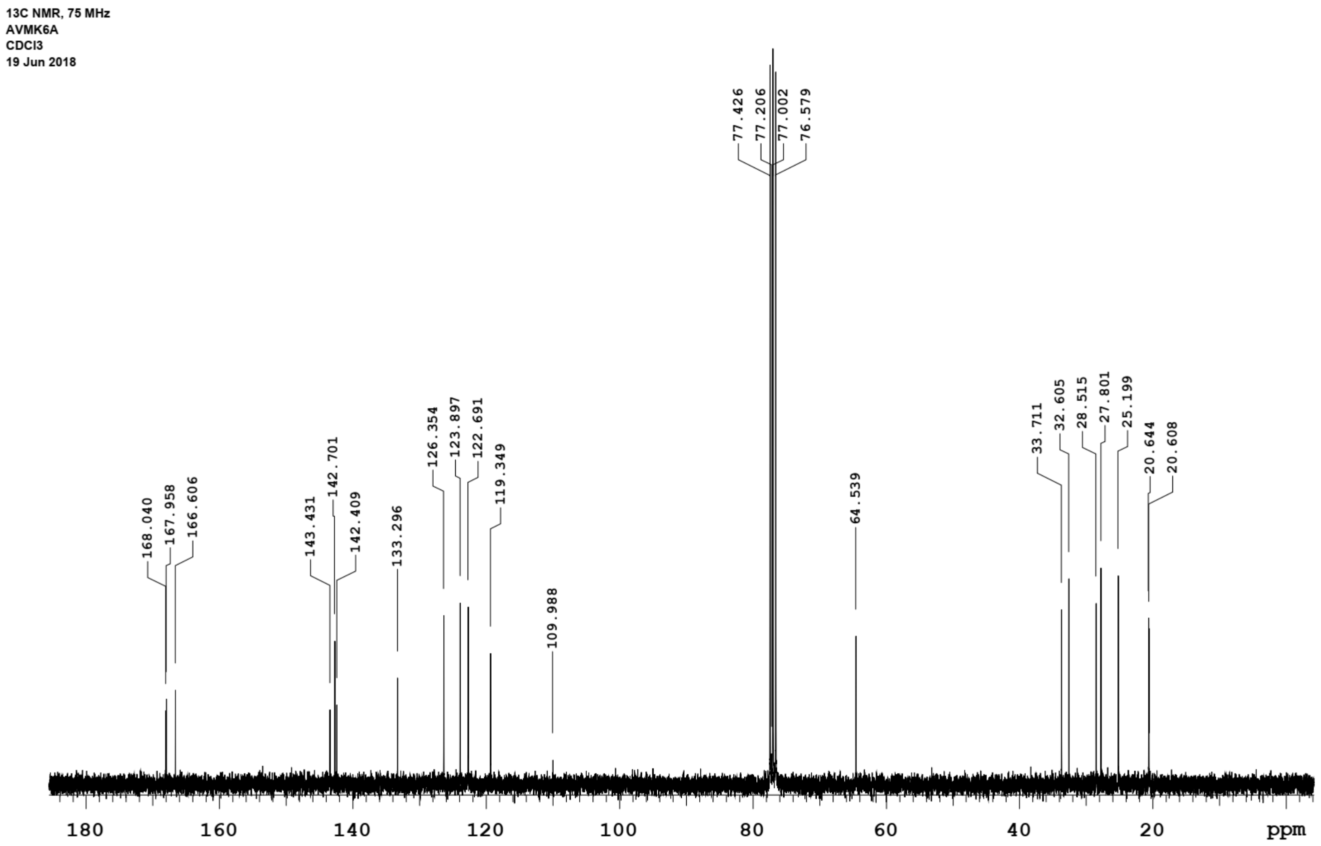

Figure S14. ^{13}C NMR data of CAP 6 ester (in $CDCl_3$)

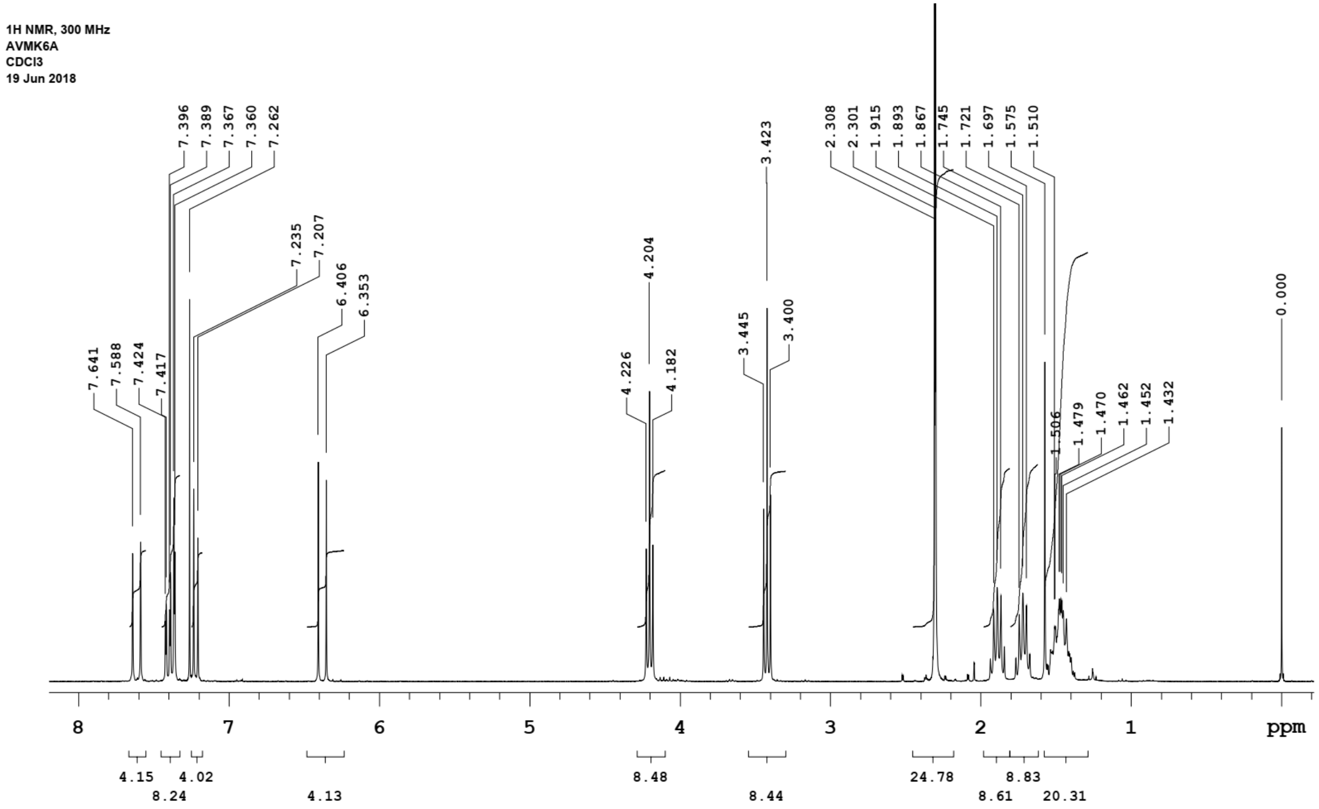

Figure S15. ^{1}H NMR data of CAP 6 ester (in $CDCl_3$)

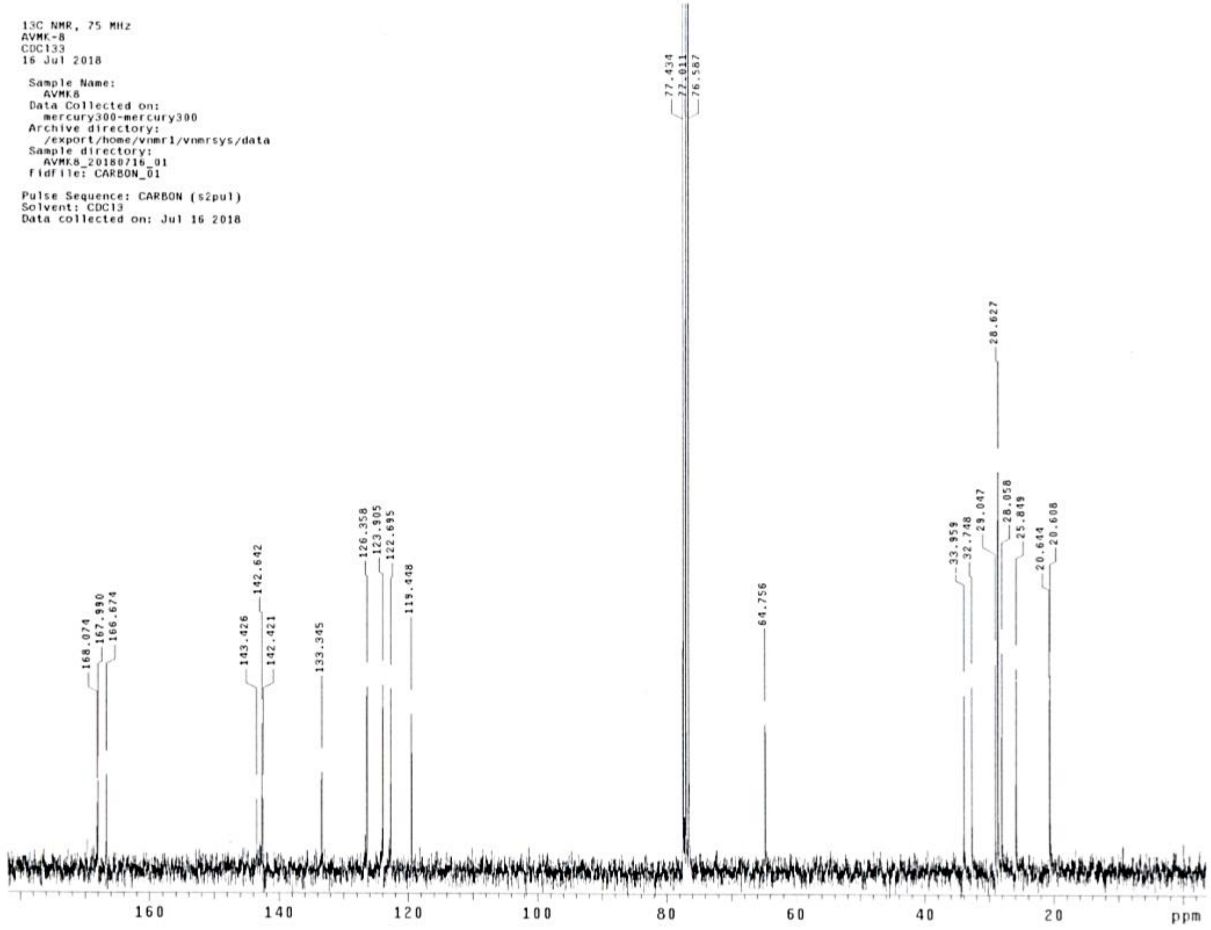

Figure S16. ^{13}C NMR data of CAP 8 ester (in $CDCl_3$)

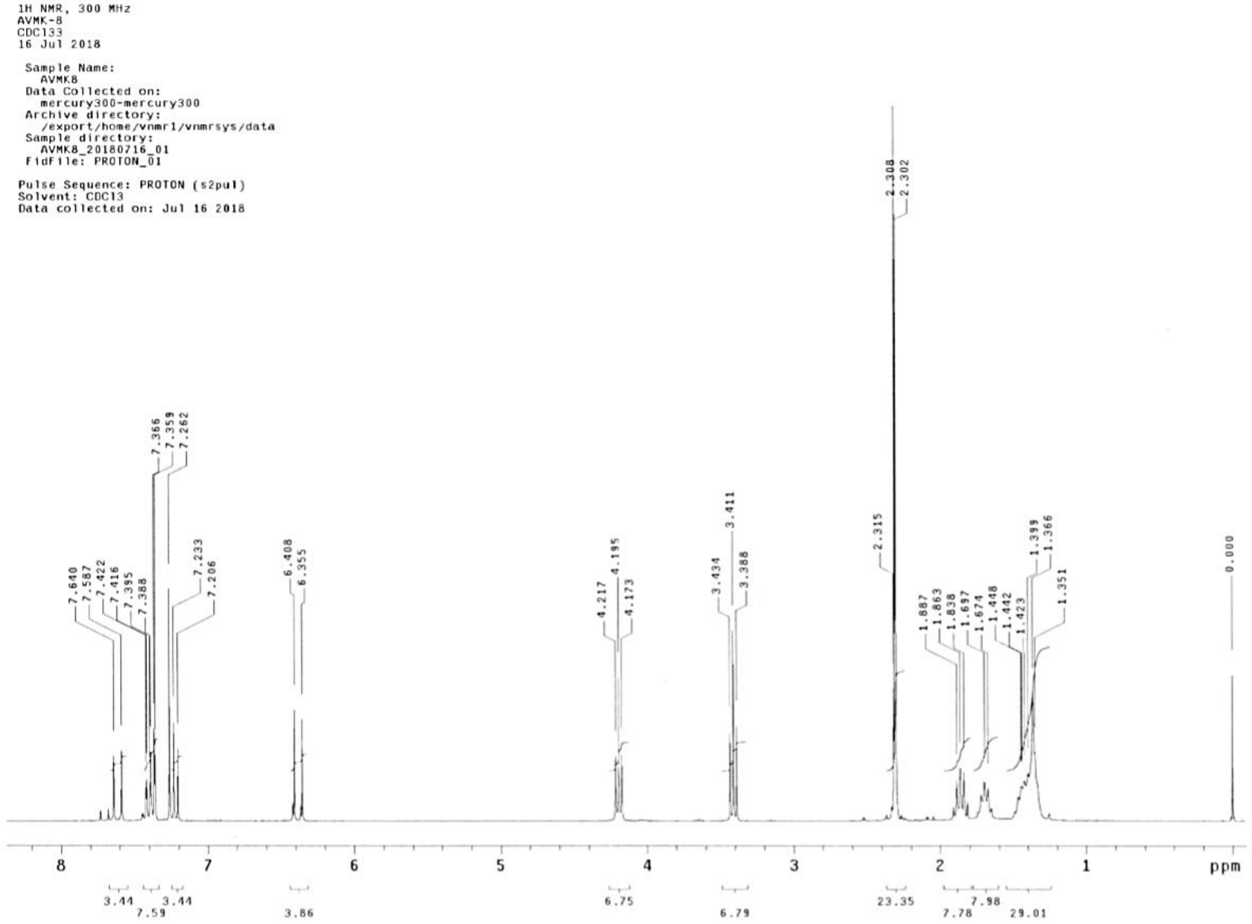

Figure S17. ^{1}H NMR data of CAP 8 ester (in $CDCl_3$)

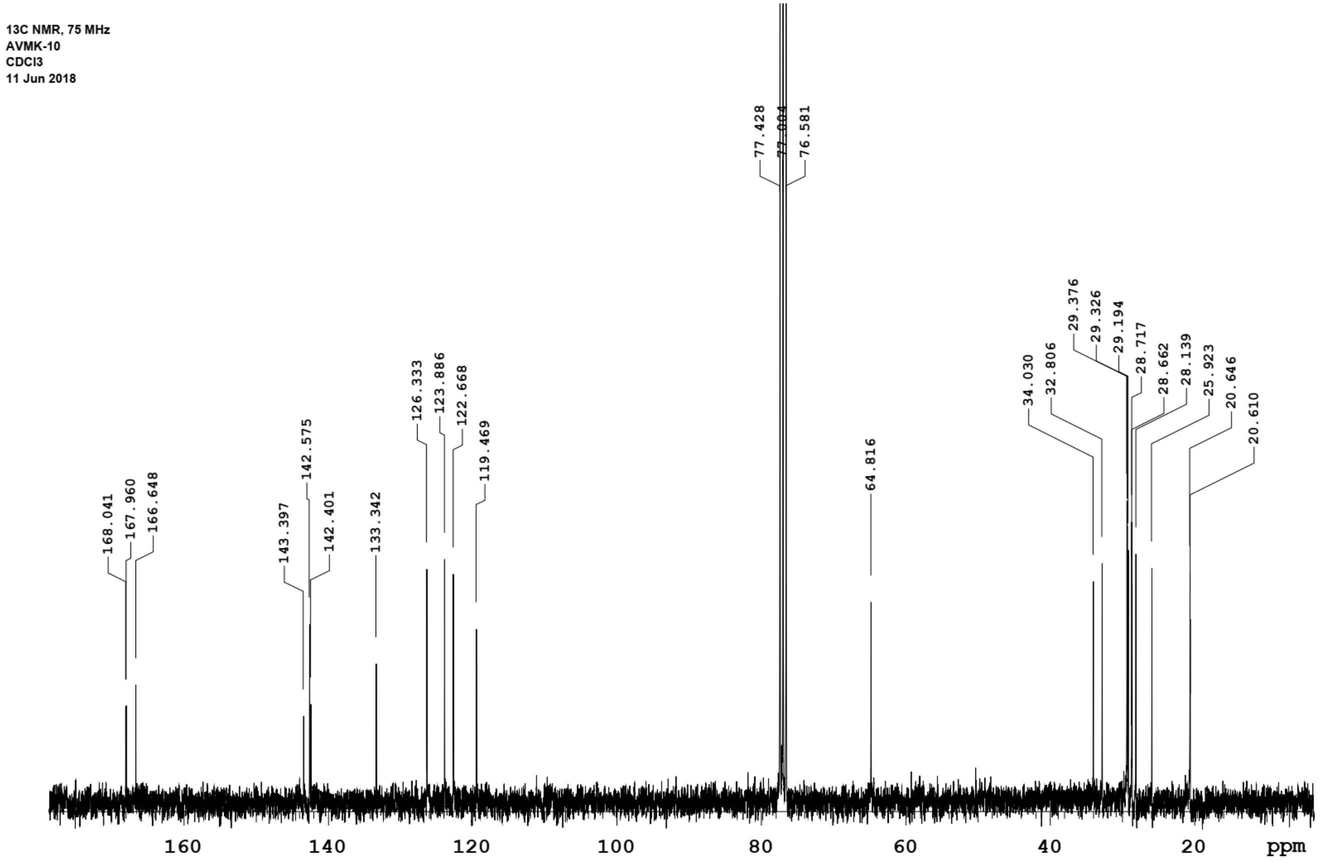

Figure S18. ^{13}C NMR data of CAP 10 ester (in $CDCl_3$)

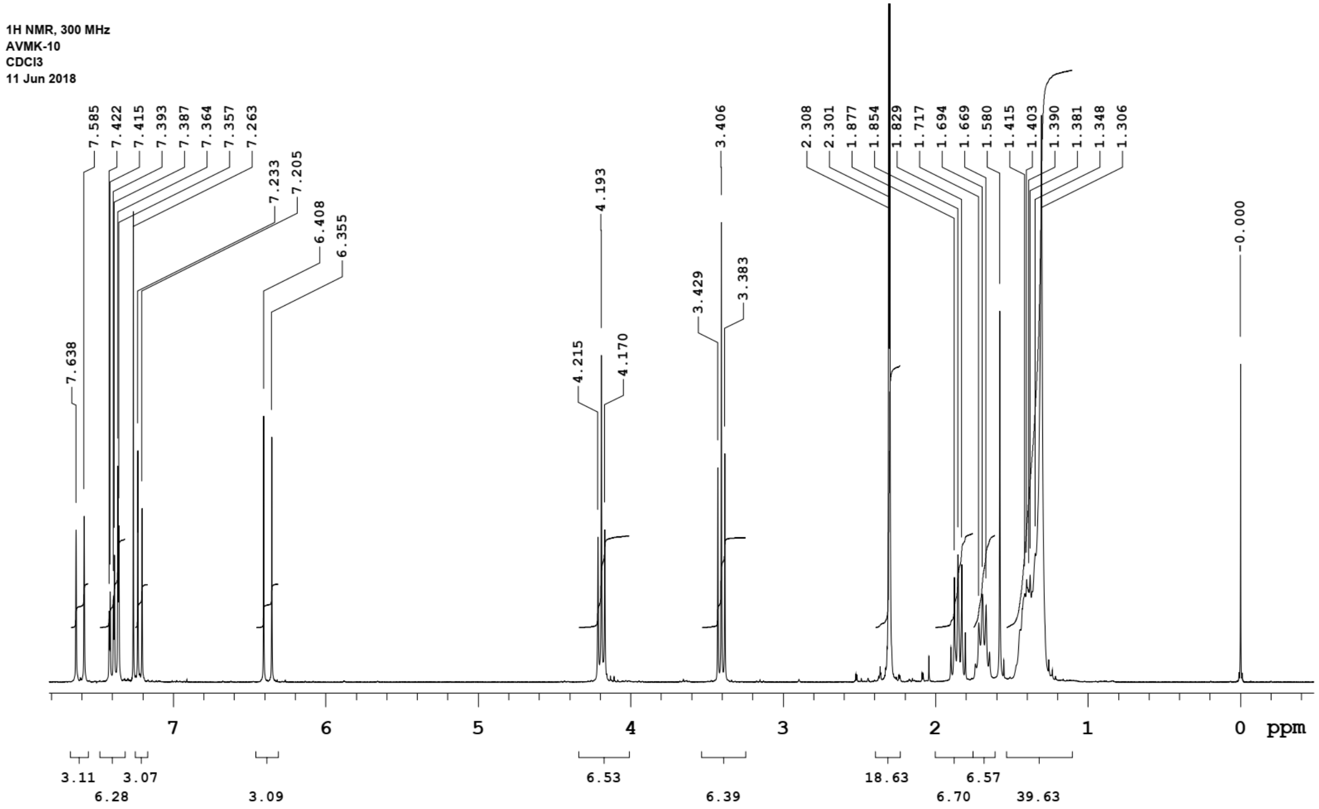

Figure S19. ^{1}H NMR data of CAP 10 ester (in $CDCl_3$)

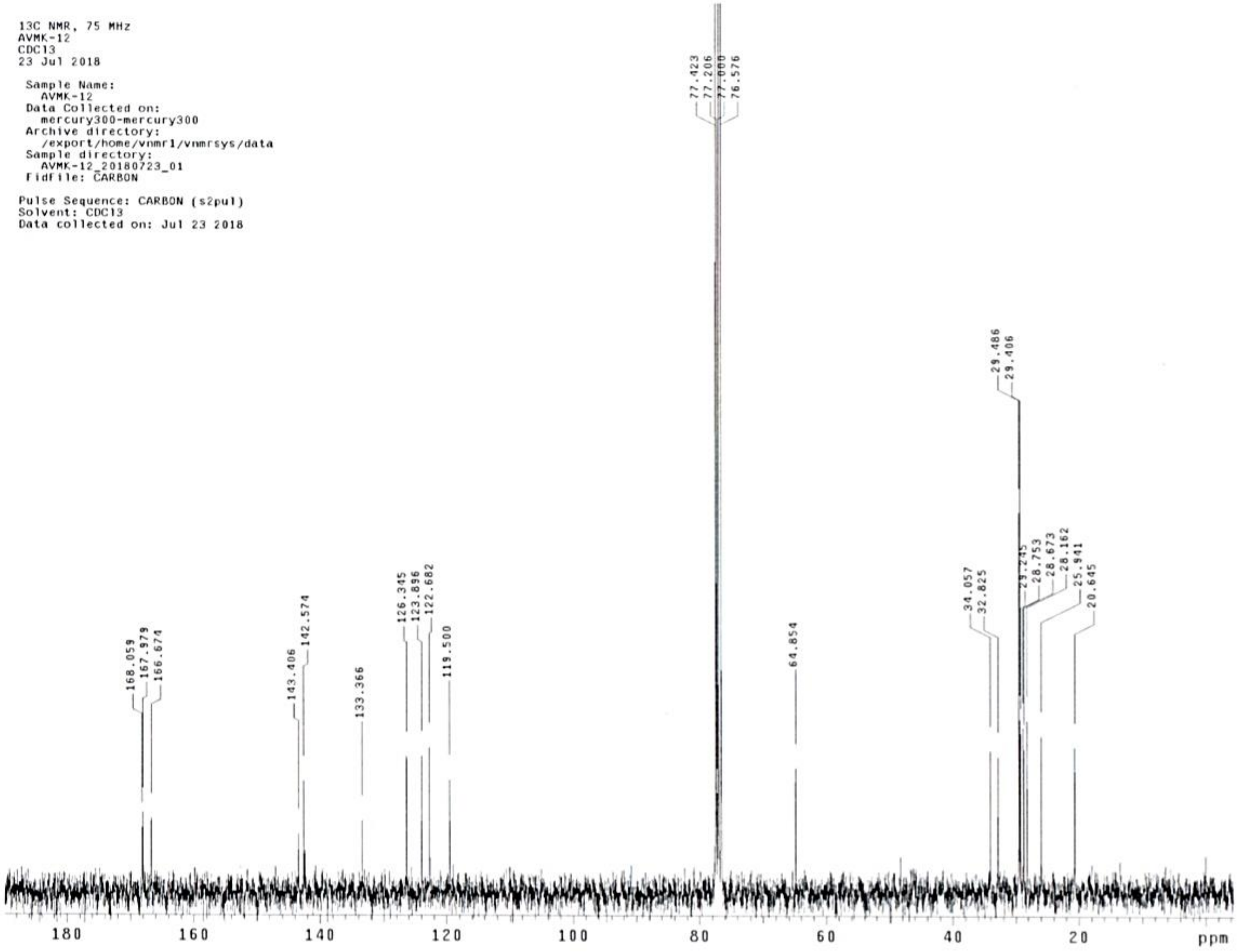

Figure S20. ^{13}C NMR data of CAP 12 ester (in $CDCl_3$)

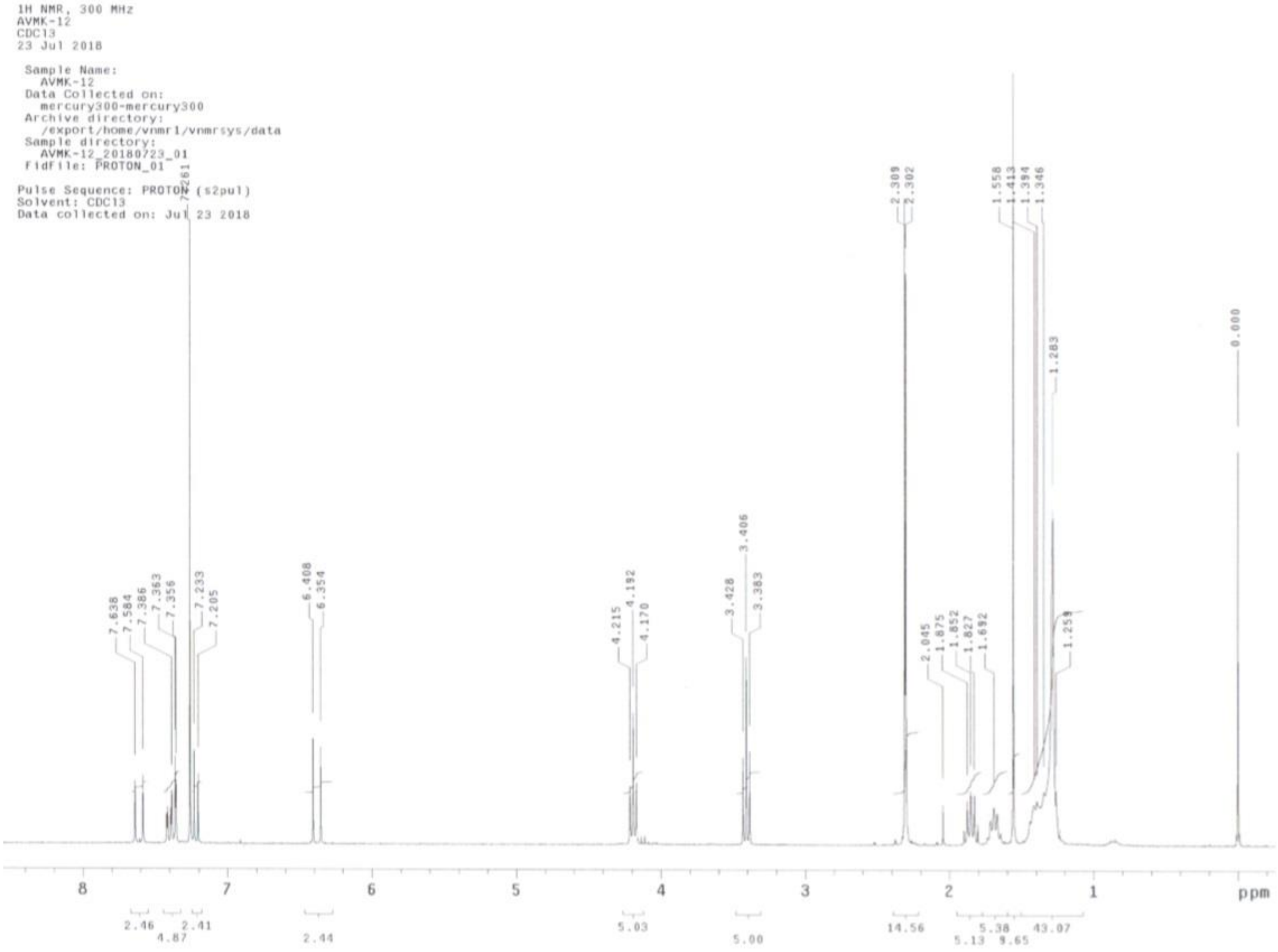

Figure S21. ^{1}H NMR data of CAP 12 ester (in $CDCl_3$)

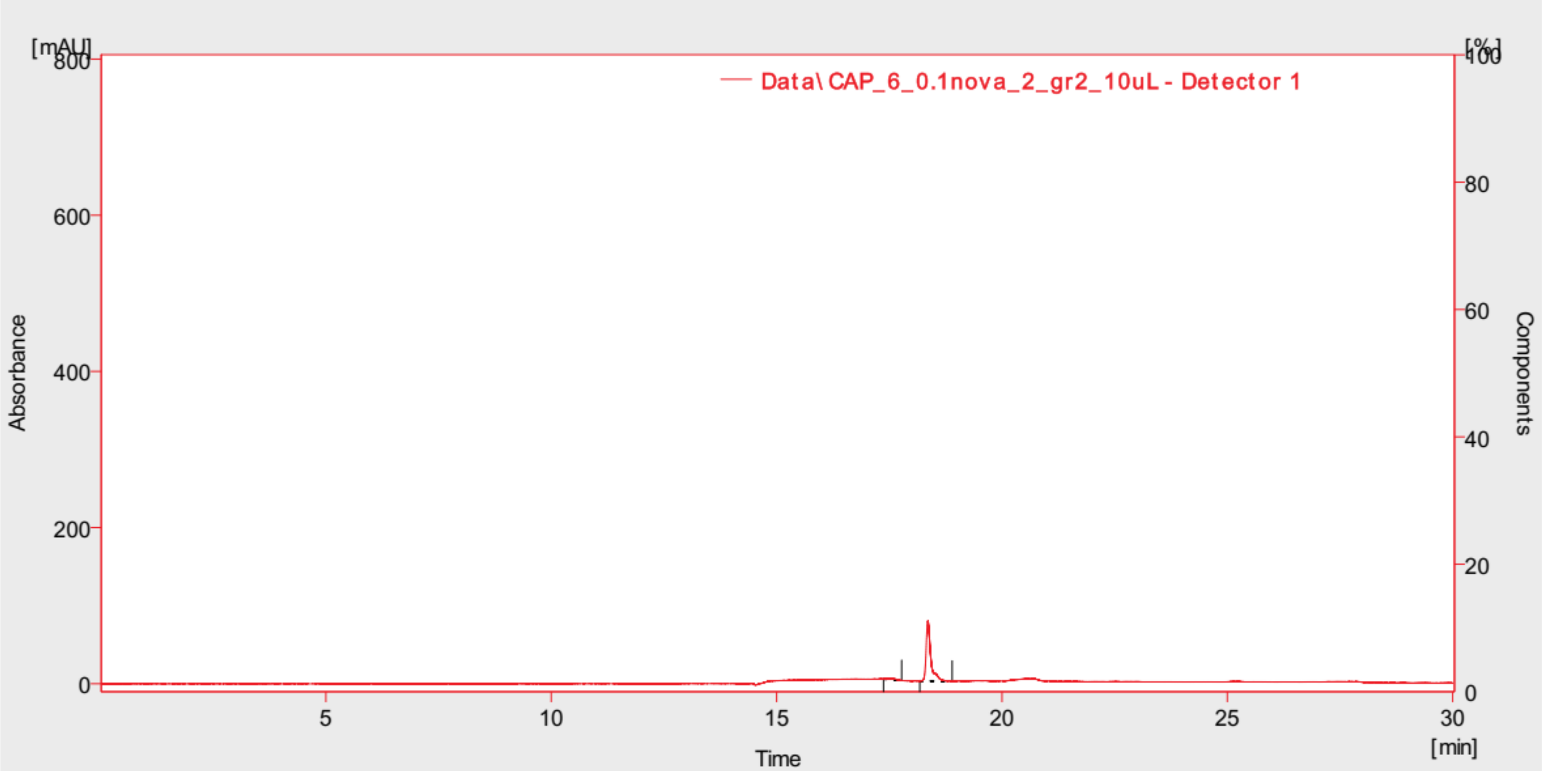

Result Table (Uncal - Data\CAP_6_0.1nova_2_gr2_10uL - Detector 1)

	Reten. Time [min]	Area [mAU.s]	Height [mAU]	Area [%]	Height [%]	W05 [min]	PDA Peak Purity	Compound Name
1	17,463	18,987	1,039	3,3	1,3	0,22	1000	
2	18,357	554,007	77,056	96,7	98,7	0,09	988	
	Total	572,994	78,096	100,0	100,0			

Figure S22. HPLC-DAD data of CAP 6 (60% H_2O + 0.05% TFA/40% ACN + 0.05% TFA) at λ = 254 nm

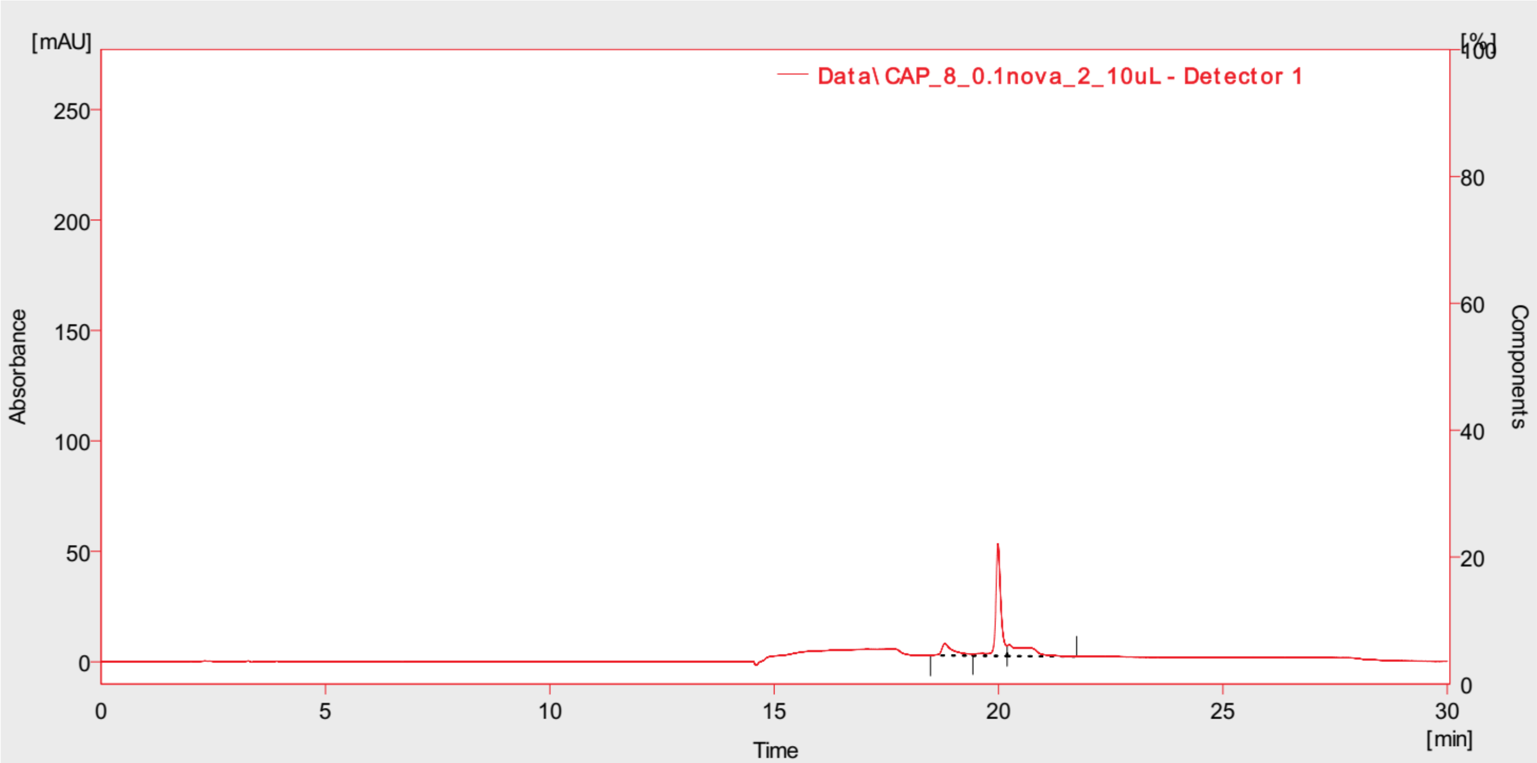

Result Table (Uncal - Data\CAP_8_0.1nova_2_10uL - Detector 1)

	Reten. Time [min]	Area [mAU.s]	Height [mAU]	Area [%]	Height [%]	W05 [min]	PDA Peak Purity	Compound Name
1	18,804	102,112	5,413	14,7	8,8	0,22	990	
2	19,989	415,533	50,786	59,9	82,8	0,11	986	
3	20,237	176,178	5,120	25,4	8,4	0,65	995	
	Total	693,824	61,320	100,0	100,0			

Figure S23. HPLC-DAD data of CAP 8 (60% H_2O + 0.05% TFA/40% ACN + 0.05% TFA) at λ = 254 nm

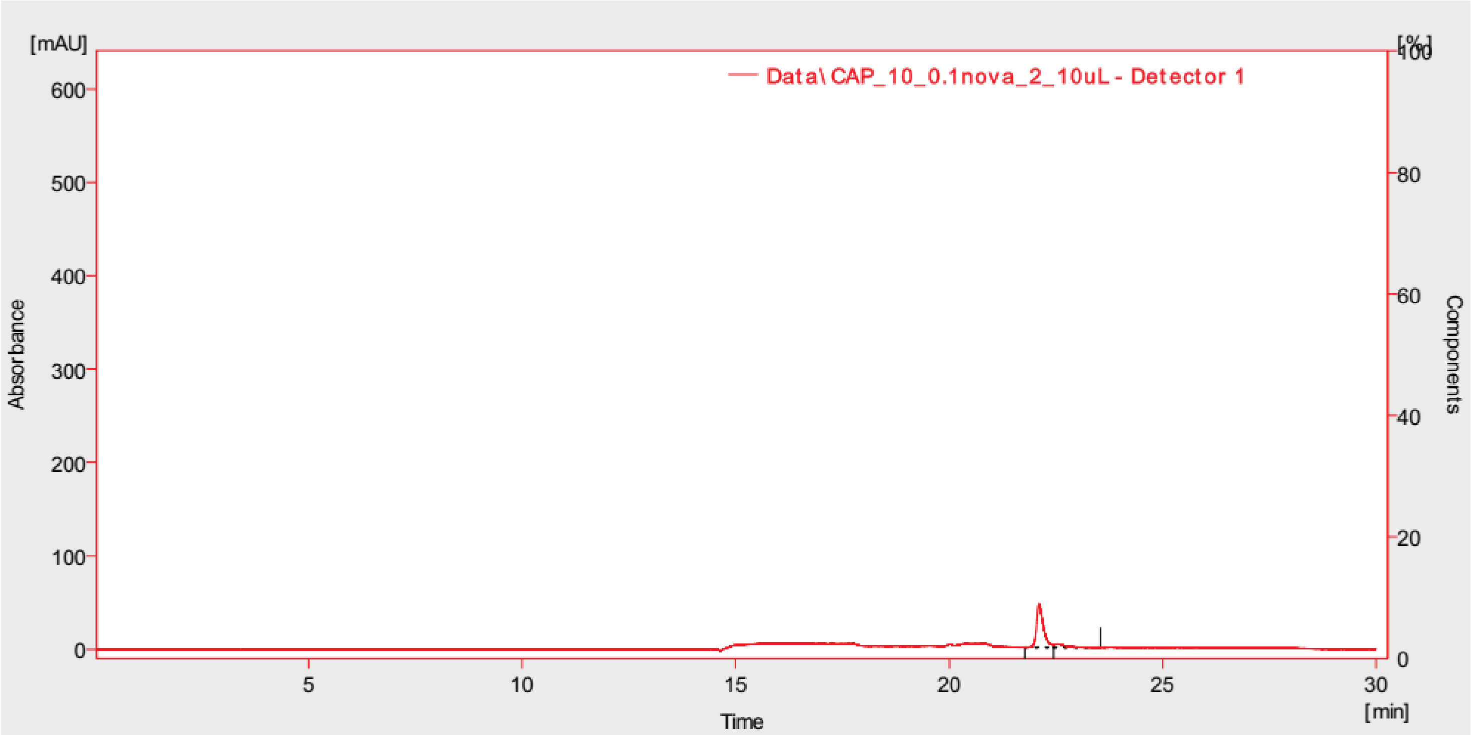

Result Table (Uncal - Data\CAP_10_0.1nova_2_10uL - Detector 1)

	Reten. Time [min]	Area [mAU.s]	Height [mAU]	Area [%]	Height [%]	W05 [min]	PDA Peak Purity	Compound Name
1	22,105	514,811	46,736	88,3	92,8	0,15	984	
2	22,561	68,292	3,617	11,7	7,2	0,25	997	
	Total	583,103	50,353	100,0	100,0			

Figure S24. HPLC-DAD data of CAP 10 (60% H_2O + 0.05% TFA/40% ACN + 0.05% TFA) at λ = 254 nm

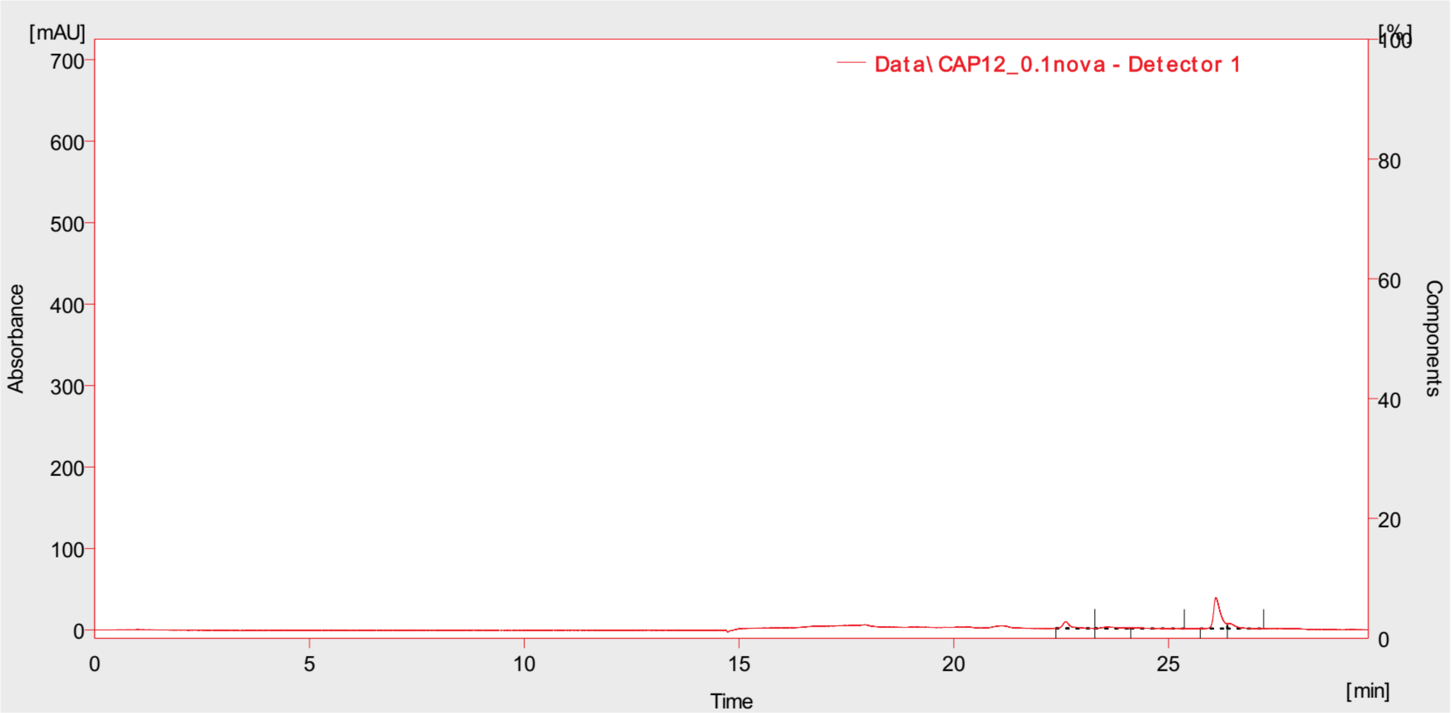

Result Table (Uncal - Data\CAP12_0.1nova - Detector 1)

	Reten. Time [min]	Area [mAU.s]	Height [mAU]	Area [%]	Height [%]	W05 [min]	PDA Peak Purity	Compound Name
1	22,604	102,228	8,380	14,9	15,1	0,15	987	
2	23,583	48,823	1,725	7,1	3,1	0,44	998	
3	24,273	21,812	0,918	3,2	1,7	0,26	999	
4	26,100	431,825	38,151	62,8	68,8	0,16	993	
5	26,429	83,343	6,269	12,1	11,3	0,18	991	
	Total	688,031	55,444	100,0	100,0			

Figure S25. HPLC-DAD data of CAP 12 (60% H_2O + 0.05% TFA/40% ACN + 0.05% TFA) at λ = 254 nm
